# Frequencies, Laboratory Features, and Granulocyte Activation in Chinese Patients with *CALR*-Mutated Myeloproliferative Neoplasms

**DOI:** 10.1371/journal.pone.0138250

**Published:** 2015-09-16

**Authors:** Haixiu Guo, Xiuhua Chen, Ruiyuan Tian, Jianmei Chang, Jianlan Li, Yanhong Tan, Zhifang Xu, Fanggang Ren, Junxia Zhao, Jie Pan, Na Zhang, Xiaojuan Wang, Jianxia He, Wanfang Yang, Hongwei Wang

**Affiliations:** 1 Institute of Hematology, the Second Hospital of Shanxi Medical University, Taiyuan, China; 2 Department of Microbiology and Immunology, School of Basic Medicine, Shanxi Medical University, Taiyuan, China; B.C. Cancer Agency, CANADA

## Abstract

Somatic mutations in the *CALR* gene have been recently identified as acquired alterations in myeloproliferative neoplasms (MPNs). In this study, we evaluated mutation frequencies, laboratory features, and granulocyte activation in Chinese patients with MPNs. A combination of qualitative allele-specific polymerase chain reaction and Sanger sequencing was used to detect three driver mutations (i.e., *CALR*, *JAK2*V617F, and *MPL*). *CALR* mutations were identified in 8.4% of cases with essential thrombocythemia (ET) and 5.3% of cases with primary myelofibrosis (PMF). Moreover, 25% of polycythemia vera, 29.5% of ET, and 48.1% of PMF were negative for all three mutations (*JAK2*V617F, *MPL*, and *CALR*). Compared with those patients with *JAK2*V617F mutation, *CALR*-mutated ET patients displayed unique hematological phenotypes, including higher platelet counts, and lower leukocyte counts and hemoglobin levels. Significant differences were not found between Chinese PMF patients with mutants *CALR* and *JAK2*V617F in terms of laboratory features. Interestingly, patients with *CALR* mutations showed markedly decreased levels of leukocyte alkaline phosphatase (LAP) expression, whereas those with *JAK2*V617F mutation presented with elevated levels. Overall, a lower mutant rate of *CALR* gene and a higher triple-negative rate were identified in the cohort of Chinese patients with MPNs. This result indicates that an undiscovered mutant gene may have a significant role in these patients. Moreover, these pathological features further imply that the disease biology varies considerably between mutants *CALR* and *JAK2*V617F.

## Introduction

The three classic *BCR-ABL*-negative myeloproliferative neoplasms (MPNs) include polycythemia vera (PV), essential thrombocythemia (ET), and primary myelofibrosis (PMF). In early 2005, a unique gain-of-function mutation in the Janus kinase 2 gene (*JAK2*V617F) was identified in approximately 95% of patients with PV and in 50% to 60% of patients with ET or PMF [1,2]. Subsequently, somatic mutations of *JAK2* exon 12 are found in most PV patients without *JAK2*V617F mutation [3,4], whereas the thrombopoietin receptor (*MPL*) mutations are present in a small number of patients with ET or PMF [5,6]. The discovery of these mutations has greatly facilitated the diagnosis of the three disorders. However, approximately one-third of patients with ET or PMF do not carry *JAK2* or *MPL* mutation, and the molecular basis of these neoplasms remains unknown.

Somatic mutations in the calreticulin (*CALR*) gene were detected in about 25% to 35% of all patients with ET and PMF [7,8]. These mutations are mutually exclusive with mutations in both *JAK2* and *MPL*. More than 50 different types of mutations in *CALR* have been detected to date, but the 52 bp deletion (type 1 mutation, c.1179_1230del) and the 5 bp insertion (type 2 mutation, c.1234_1235insTTGTC) are the most frequent. Overall, these mutations are found in 80% to 90% of all tested patients with mutant *CALR*. All reported *CALR* mutations result in a frameshift, owing to insertions or deletions in the last exon (exon 9). Functional analysis showed that overexpression of the most frequent *CALR* deletion can cause cytokine-independent growth in vitro and result in the activation of STAT5 through an unknown mechanism [7,8]. These data suggest that *CALR* mutations play a causal role during the development and evolution of MPNs, similar to *JAK2* and *MPL* mutations.

In this study, we investigated the profile and laboratory features of *CALR* mutations in Chinese patients with MPN. We also compared the differences between the present findings and those from four different geographical sites in China and Western countries. To further explore the biological effects of *CALR* mutations, we analyzed the leukocyte alkaline phosphatase (LAP) characteristics of the MPN patients with *CALR* mutations. This study is the first to report the relationship between *CALR* mutations and LAP expression in MPN patients.

## Materials and Methods

### Ethics statement

This study complied with the Declaration of Helsinki and was approved by the Ethics Committee of Shanxi Medical University. Written informed consent was obtained from all patients and from the legal guardians in the case of minors.

### Patient samples

A total of 668 MPN patients, including 128 PV, 407 ET, and 133 PMF, were diagnosed according to the World Health Organization criteria [9]. The relevant diagnosis criteria for *JAK2* mutation-negative PV included bone marrow histology, serum Epo levels, and endogenous erythroid colonys (EECs) [9]. The patients were recruited from the Department of Hematology, the Secondary Hospital of Shanxi Medical University, Shanxi Province, China. These patients had stored samples of granulocyte DNA from bone marrow or peripheral samples. The patients’ laboratory features, including age, gender, and hematological parameters, were obtained from the medical records at diagnosis. LAP expression was evaluated by LAP score. The patients’ LAP scores were obtained from the medical records at diagnosis and were tested through routine clinical testing conducted in our laboratory. LAP scores were measured using the LAP staining kit (Sunny Biotechnology, Shanghai, China) according to the manufacturer’s instructions. The procedure involves counting 100 neutrophils, including lobed and band forms, but excluding other left-shifted granulocytes, eosinophils, and basophils. The reaction was scored from 0 to 4 depending on the number of stained granules and the intensity of the stain. The number of cells was multiplied by the score and added up to a normal range (40–80). Puerperal is applied to positive contrast.

### *JAK2*V617F, *MPL*, and *CALR* mutation assays

Allele-specific PCR (AS-PCR) was introduced for the screening of *JAK2*V617F point mutation. The reverse primer (V617F primer: 5′-GTTTTACTTACTCTCGTCTCCACAAAA-3′) of AS-PCR was specific for the mutant allele and contains an intentional mismatch at the last nucleotide from the 3′ end. The outer primer pairs (forward primer: 5′-CCTCAGAACGTTGATGGCA-3′, reverse primer: 5′-ATTGCTTTCCTTTTTCACAAGA-3′) were designed to amplify fragments from the V617F mutant and wild-type alleles as internal control. The PCR product was analyzed using an ethidium bromide-impregnated 2% agarose gel electrophoresis. The electrophoresis result showed a 295 bp mutant-type band and a 451 bp wild-type band.

Genomic PCR amplification combined with directional Sanger sequencing was applied to screen *CALR* and *MPL* mutations. A pair of oligonucleotide primers covering exon 9 of *CALR* was used to amplify a 1019 bp product (forward: 5′-AAACCCTGTCCAAAGCAAG-3′ and reverse: 5′-GGAGACACAAAATTTAATTTAATAG-3′). Oligonucleotide primers targeting *MPL* exon 10 were employed to amplify a 218 bp product (forward: 5′-TAGGGGCTGGCTGGATGAG-3′ and reverse: 5′-CTTCGGCTCCACCTGGTCC-3′). PCR products were purified and subjected to directional sequencing.

### Statistical analysis

Statistical analyses were performed using SPSS 13.0 software. Numerical variables were summarized by median and range, and categorical variables by count and relative frequency (%) of each category. The parameters between patient groups were statistically analyzed with the nonparametric Wilcoxon rank-sum test (for measurement data) and the Fisher’s exact test or Pearson Chi-square test (for enumeration data). *P* < 0.05 was considered statistically significant (two-tailed).

## Results

### Mutational frequencies of *CALR* gene in patients with MPN

Three mutations, namely, *JAK2*V617F, *MPL*, and *CALR*, were simultaneously screened in a cohort of 668 Chinese patients with confirmed PV (N = 128), ET (N = 407), and PMF (N = 133) from our center to estimate *CALR* mutation profile in Chinese patients with MPN. As shown in Table 1, *JAK2*V617F mutation was detected in 75.0% of PV, 61.2% of ET, and 45.1% of PMF. *MPL* exon 10 mutations were identified in six patients. *MPL*W515L was found in two cases with ET and one case with PMF; *MPL*W515K was detected in one case with PMF; and *MPL*A497-L498LVIAins was found in two cases with ET. *MPL*A497-L498LVIAins is an insertion mutation previously reported by our group [10]. *CALR* mutations were identified in 34 patients with ET and 7 with PMF, accounting for 8.4% of cases with ET and 5.3% of cases with PMF, respectively. In our cohort, none of the patients with PV were found harboring *MPL* and *CALR* mutations. The three mutations (*JAK2*V617F, *MPL*, and *CALR*) were mutually exclusive in patients with ET and PMF. Notably, 29.5% of ET and 48.1% of PMF patients showed no mutations on all three genes (triple-negative).

**Table 1:** Frequencies and distribution of *JAK2*V617F, *MPL* exon 10, and *CALR* exon 9 mutations in PV, ET, and PMF.

		Different regions of China					Western countries			
		Our group(Shanxi)	*Li* et al. [11](Tianjin)	*Shen* et al. [12](Jiangsu)	*Qiao* et al. [13](Nanjin)	*Chen* et al. [14](Taiwan)	*Nangalia* et al. [8](UK)	*Klampfl* et al. [7](Austrian)	*Rumi* et al. [15,16](Italy)	*Andrikovics*[17](French)
PV	JAK2V617F^+^	75.0% (96/128)	—	—	91.9% (34/37)	—	100% (48/48)	95.0% (363/382)	96% (468/490)	—
	MPL exon 10^+^	0 (0/128)	—	—	0 (0/37)	—	0 (0/48)	0 (0/382)	0 (0/490)	—
	CALR exon 9^+^	0 (0/128)	—	—	0 (037)	—	0 (0/48)	0 (0/382)	0 0/490)	—
	Triple-negative	25.0% (32/128)	—	—	8.1% (3/37)	—	0 (0/48)	5.0% (19/382)	4% (22/490)	—
ET	JAK2V617F^+^	61.2%(249/407)	—	51.1% (97/190)	45.5%(101/222)	63.9% (94/147)	56.0% (35/62)	59.2% (184/311)	22% (466/745)	53.0% (154/289)
	MPL exon 10^+^	1.0% (4/407)	—	3.2% (6/190)	0.9% (2/222)	2.7% (4/147)	8.0% (5/62)	3.5% (11/311)	4% (28/745)	3.0% (9/289)
	CALR exon 9^+^	8.4% (34/407)	—	26.3% (50/190)	31.1% (69/222)	22.5% (33/147)	28.0% (17/62)	25.1% (78/311)	24% (176/745)	33.0% (96/289)
	Triple-negative	29.5% (120/407)	—	19.4% (38/190)	22.5% (50/222)	10.9% (16/147)	8.0% (5/62)	12.2% (38/311)	10% (75745)	11.0% (30/289)
PMF	JAK2V617F^+^	45.1% (60/133)	50.0%(178/357)	—	60.6% (20/33)	—	69.0% (35/62)	53.2% (108/203)	64.7% (399/617)	57.0% (56/99)
	MPL exon 10^+^	1.5% (2/133)	3.0% (11/357)	—	0 (0/33)	—	5.0% (2/62)	6.4% (13/203)	4.0% (25/617)	7.0% (7/99)
	CALR exon 9^+^	5.3% (7/133)	21.0% (76/357)	—	12.1% (4/33)	—	23.0% (9/62)	35.5% (72/203)	22.7% (140/617)	25.0% (25/99)
	Triple-negative	48.1% (64/133)	27.0% (96/357)	—	27.5% (9/33)	—	3.0% (1/62)	4.9% (10/203)	8.6% (53/617)	11.0% (11/99)

“Triple-negative” represents patients without *JAK2*V617F, *MPL*, and *CALR* mutations. “—” indicates no detection performed in corresponding studies.

For mutation types, 12 distinct variants of *CALR* mutations were identified in our cohort, including 6 deletions (c.1179_1230del, c.1174_1219del, c.1170_1221del, c.1185_1218del, c.1196_1226del, and c.1179_1221del), 4 insertions (c.1234_1235insTTGTC, c.1235_1236insTGTCG, c.1237_1238insAAGGA, and c.1228_1229insTCCTTGTC), and 2 complex indels (c.1179_1233delinsAGG and c.1200_1220delinsTCTTGTCT). Among these mutation types, c.1179_1230del (mutation type 1, a 52 bp deletion) and c.1234_1235insTTGTC (mutation type 2, a 5 bp insertion) were the most frequent alterations, representing 51.2% and 24.4% of cases with mutant *CALR*, respectively. The remaining 10 types were previously unidentified, novel *CALR* mutations. Consistent with the previously described *CALR* mutations, all novel mutant variants caused a +1 bp frameshift and led to a novel C-terminal peptide sequence. These novel mutations were only observed in a single ET patient. In ET patients with mutant *CALR*, type 1 mutations were the most common (41.2%), followed by type 2 (26.5%), and the other 10 novel mutation types were found in one ET patient (2.9%). In PMF patients with mutant *CALR*, only types 1 (85.7%) and 2 (14.3%) were detected (Table 2).

**Table 2:** Comparison analysis of MPN phenotypes between types 1 and 2 mutations in *CALR* gene.

		Different regions of China					Western countries	
		Our group	*Qiao* et al.	*Chen* et al.	*Shen* et al.	*Li* et al.	*Nangalia* et al.	*Klampfl* et al.
ET cohort	Type 1	41.2% (14/34)	44.9% (31/69)	54.5% (18/33)	46.0% (23/50)	—	38.8% (38/98)	46.2% (90/195)
	Type 2	26.5% (9/34)	33.3% (23/69)	27.3% (9/33)	30.0% (15/50)	—	48.0% (47/98)	37.9% (74/195)
PMF cohort	Type 1	85.7% (6/7)	25.0% (1/4)	—	—	31.6% (24/76)	60.5% (23/38)	65.7% (69/105)
	Type 2	14.3% (1/7)	50.0% (2/4)	—	—	64.5% (49/76)	23.7% (9/38)	20.0% (21/105)

Type 1: c.1179_1230del; Type 2: c.1234_1235insTTGTC; “—” indicates no detection performed in the corresponding studies.

### Demographic and laboratory features of ET or PMF patients stratified by *CALR* and *JAK2*V617F mutations

Medical records at diagnosis of Chinese patients with *CALR* or *JAK2*V617F mutations were collected to define their demographic and laboratory features. Only 267 patients with ET and 62 patients with PMF had complete records. Among patients with ET, those with *CALR* mutations (*CALR*+) showed lower hemoglobin (Hb) levels (*P* < 0.001), lower white blood cell (WBC) counts (*P* < 0.001), and higher platelet (PLT) counts (*P* = 0.003) than those with *JAK2*V617F mutation (*JAK2*V617F+) (Table 3). Frequencies for age and gender were balanced between the two groups (Table 3). For different CALR mutation types in ET patients, statistical difference was not observed in age, gender, platelet counts, Hb levels, and WBC counts between *CALR* type1+ group and *CALR* type2+ group (Table 4). Compared with patients with *JAK2*V617F+ (Table 4), patients with *CALR* type1+ presented lower WBC counts (*P* = 0.003). Male patients with *CALR* type1+ showed higher Hb levels (*P* = 0.025) than those with *JAK2*V617F+, whereas female patients with *CALR* type1+ showed lower Hb levels (*P* = 0.000) than ones with *JAK2*V617F+. No statistical differences were observed for age, gender, and platelet counts between patients with *CALR* type1+ and *JAK2*V617F+. Patients with *CALR* type2+ exhibited lower WBC counts (P = 0.002) and higher platelet counts (P = 0.002) than patients with *JAK2*V617F+ (Table 4). No statistical differences were showed for Hb levels between male patients with *CALR* type2+ and *JAK2*V617F+, whereas female patients with *CALR* type2+ presented lower Hb levels (P = 0.003) than female patients with *JAK2*V617F+. Significant differences were not found in age and gender between patients with *CALR* type2+ and *JAK2*V617F+. Similarly, *CALR*+ and *JAK2*V617F+ shared similar laboratory features in patients with PMF. These groups showed no significant differences in age, gender, Hb levels, and WBC and PLT counts (Table 3). Among PMF patients with different *CALR* mutation types, only one patient with *CALR* type2+ had a complete record. Hence, comparison analysis was only conducted in *CALR* type1+ and *JAK2*V617F+ patients. No significant differences were noted in age, gender, Hb levels, and WBC and PLT counts between *CALR* type1+ and *JAK2*V617F+ patients (Table 5). Laboratory features of the PMF patient with *CALR* type2+ are described in Table 5.

**Table 3:** Demographic and laboratory features at diagnosis of ET and PMF patients with *CALR* and *JAK2*V617F mutations.

	ET cohort		PMF cohort		*P* value	
	(A) *CALR* ^+^(*N* = 29)	(B) *JAK2*V617F^+^(*N* = 238)	(C) *CALR* ^+^(*N* = 6)	(D) *JAK2*V617F^+^(*N* = 56)	(A) vs (B)	(C) vs (D)
Age, median (range)(years)	59 (15–90)	58 (23–78)	63(53–71)	60 (22–81)	*P* = 0.872	*P* = 0.957
Sex (male:female)	17:12	108:130	2:4	25:31	*P* = 0.177	*P* = 0.689
WBC count, median (range)(×10^9^/L)	12.2 (4.1–37)	15.4 (4.1–77.1)	5.2 (2.6–10.2)	15.9 (0.5–110.0)	*P* < 0.001	*P* = 0.096
Hb, median (range)(g/L)	123.8 (66.4–172.0)	145.4 (52.6–428)	116.9 (70.0–168.0)	111.3 (47.6–169.0)	*P* < 0.001	*P* = 0.710
PLT count, median (range)(×10^9^/L)	1023 (420–2640)	764 (404–2274)	204 (115–254)	177 (18–383)	*P* = 0.003	*P* = 0.482

WBC: white blood cell; Hb: hemoglobin; PLT: platelet.

**Table 4:** Demographic and laboratory features at diagnosis of ET patients with different types of *CALR* and *JAK2*V617F mutations.

	ET cohort			*P* value		
	(A) *CALR*-type1^+^(*N* = 14)	(B) *CALR*-type2^+^(*N* = 8)	(C) *JAK2*V617F^+^(*N* = 238)	(A) vs (B)	(A) vs (C)	(B) vs (C)
Age, median (range) (years)	55 (24–72)	57 (31–78)	58 (23–78)	*P* = 0.804	*P* = 0.515	*P* = 0.858
Sex (male:female)	9:5	5:3	108:130	*P* = 1.000	*P* = 0.168	*P* = 0.476
WBC count, median (range) (×10^9^/L)	10.7 (4.4–37.0)	8.4 (6.7–12.2)	15.4 (4.1–77.1)	*P* = 0.616	*P* = 0.003	*P* = 0.002
Hb, median (range) (g/L)						
Male	140.0 (91.3–157.0)	124.3 (111.0–172.0)	125.0 (52.6–144.0)	*P* = 0.548	*P* = 0.025	*P* = 0.402
Female	122.0 (104.0–137.0)	121.0 (118.0–134.0)	162.0 (144.0–428.0)	*P* = 0.881	*P*<0.001	*P* = 0.003
PLT count, median (range) (×10^9^/L)	900 (420–1863)	1396 (618–2640)	764 (404–2274)	*P* = 0.082	*P* = 0.078	*P* = 0.002

WBC: white blood cell; Hb: hemoglobin; PLT: platelet.

**Table 5:** Demographic and laboratory features at diagnosis of PMF patients with different types of *CALR* and *JAK2*V617F mutations.

	PMF cohort			*P* value		
	(A) *CALR*-type1^+^(*N* = 5)	(B) *CALR*-type2^+^(*N* = 1)	(C) *JAK2*V617F^+^(*N* = 56)	(A) vs (B)	(A) vs (C)	(B) vs (C)
Age, median (range)(years)	64 (53–71)	70	60 (22–81)	—	*P* = 0.841	—
Sex (male:female)	2:3	female	25:31	—	*P* = 1.000	—
WBC count, median (range)(×10^9^/L)	4.58 (2.58–10.20)	3.34	15.9 (0.5–110.0)	—	*P* = 0.103	—
Hb, median (range)(g/L)	117.0 (70.0–168.0)	95.1	111.3 (47.6–169.0)	—	*P* = 0.953	—
PLT count, median (range)(×10^9^/L)	223 (175–254)	220	177 (18–383)	—	*P* = 0.441	—

WBC: white blood cell; Hb: hemoglobin; PLT: platelet; “—” indicates no analysis performed.

### Analysis of LAP expression in PV, ET, and PMF patients with *CALR* and *JAK2*V617F mutations

**Fig 1:**
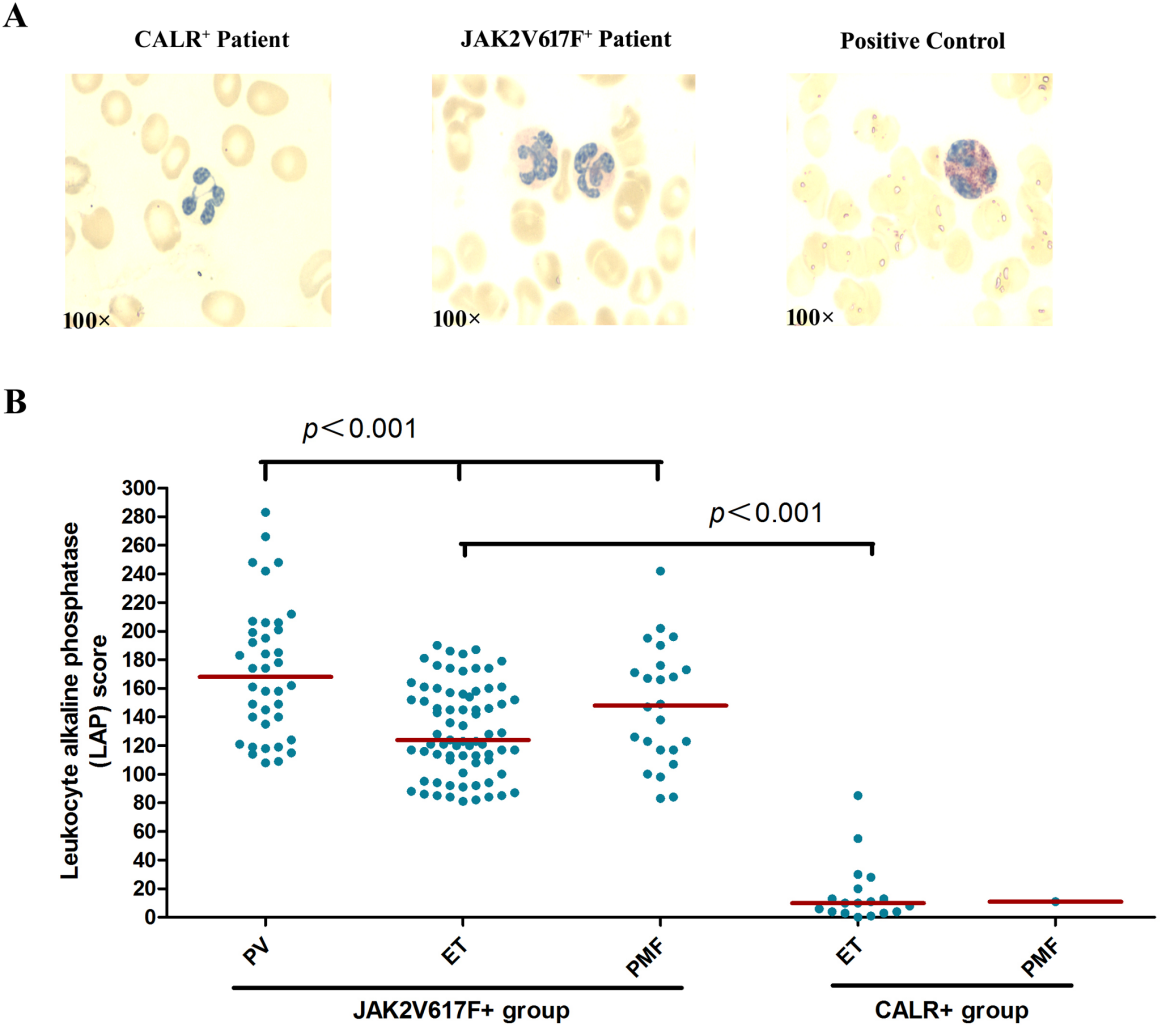
**(A) LAP scoring images of CALR and JAK2V617F mutant patients**. Peripheral blood smear of a representative CALR mutant patient displays a poor LAP expression (positive-cells:1%; score:1). In contrast, peripheral blood smear of a representative JAK2V617F mutant patient reveals a marked LAP expression (positive-cells:90%; score:194). Peripheral blood smear of a representative positive control (Puerperal) shows the highest intensity level of the stain (level: 3). **(B) Leukocyte alkaline phosphatase (LAP) scores in circulating granulocytes of peripheral blood from different categories of patients carrying *JAK2*V617F or *CALR* mutation**. LAP values are shown in a scatter plot; diamond indicates the median. (i) LAP score levels of 38 PV, 74 ET, and 24 PMF patients carrying *JAK2*V617F mutant alleles. LAP values of all patients are above normal (>80). The Kruskal–Wallis test showed significant differences between the three disorders (*P* < 0.001 for all comparisons). (ii) LAP score levels of 18 ET patients and 1 PMF patient carrying *CALR* mutant alleles. The entire data showed no significant differences between the ET and PMF groups. Both ET and PMF patients carrying the *CALR* mutation exhibited lower LAP scores than the normal value. (iii) LAP score levels of 74 ET patients with *JAK2*V617F mutant alleles and 18 ET patients carrying the *CALR* mutation. The Mann–Whitney U test showed significant differences between both groups (*P* < 0.001). (iv) LAP score levels of 24 PMF patients with *JAK2*V617F mutant alleles and 1 PMF patient carrying the *CALR* mutation. These data show that LAP score levels of PMF patients with *JAK2*V617F mutation are markedly higher than those with the *CALR* mutation.

LAP scores were evaluated in patients with *CALR* and *JAK2*V617F mutations to assess their effect on granulocyte function. LAP is a key marker for granulocyte activation. High levels of LAP indicate elevated granulocyte activation. LAP scores were obtained through routine clinical testing conducted in our laboratory; the normal reference value of LAP scores ranges from 40 to 80. LAP scores of 19 patients with *CALR* mutations (ET = 18, PMF = 1) and 136 patients with *JAK2*V617F mutation (PV = 38, ET = 74, PMF = 24) were obtained at diagnosis. During the diagnosis, none of the patients presented with fever or inflammation, or received previous cytoreductive treatment. LAP score features of the two mutations are shown in Fig 1. In the *JAK2*V617F-mutated group, all 136 patients presented higher LAP scores than the normal upper limit (>80). Furthermore, the distribution of LAP scores was investigated among patients with PV, ET, and PMF. Patients with PV presented the highest LAP level (median: 168; range: 108–283), followed by patients with PMF (median: 148; range: 83–242) and patients with ET (median: 142; range: 81–353). Thus, the Kruskal–Wallis test demonstrated significant differences in LAP expression among the three groups (*P* < 0.001 for all comparisons). In the *CALR*-mutated group, the majority of patients with ET or PMF exhibited lower LAP scores. In detail, 16 out of 18 patients with ET and 1 patient with PMF presented significantly lower LAP values than the normal. LAP scores of the other two patients with ET were equal to or slightly higher than the normal LAP score. To compare the differences in LAP expression between *CALR*-mutated and *JAK2*V617F-mutated patients, we stratified patients into two different subgroups based on disease categories: ET group and PMF group. For the ET group, the median values of LAP scores in *CALR*-mutated and *JAK2*V617F-mutated patients were 17 and 142, respectively. Statistically significant difference was observed between the two mutation groups (P < 0.001) (Fig 1B). For the PMF group, data from only one PMF patient with *CALR* mutations were collected, which exhibited a lower LAP score of 11, and the median value in patients with *JAK2*V617F was 148. Although statistical analysis of these results was not performed because of the lack of sufficient samples, an apparent difference can be observed between scores from the two mutation groups (Fig 1B).

## Discussion

Since the identification of *CALR* exon 9 mutations in patients with *JAK2*-unmutated MPN [7,8], different hematologic centers worldwide successively performed detection of *CALR* mutations in MPN patients. The differences between our findings and those from the other centers are listed in Table 1. The *CALR* mutation rate in our study was extremely lower than those in the other hematologic centers, regardless of whether the subjects come from Western countries or from the four different geographical sites in China. By contrast, the findings from the four hematologic centers in China showed close frequencies to those reported in Caucasian patients. This result suggests that ethnic differences are not apparent between Chinese and Caucasian patients with regard to the *CALR* mutation rate. From the currently available data, *CALR* mutation rates exhibited a geographical difference in China, although this difference may be affected by several factors. These factors include the diagnostic criteria and assays used. Another significant difference was noted in the percentages of triple-negative patients between the Chinese and Caucasian patients. The data obtained from the four centers in China, including those from our center but excluding those by Chen *et al*., showed that up to 20%–30% of Chinese patients with ET and 27%–48% of Chinese patients with PMF were triple-negative for the mutations. These frequencies are substantially higher than those reported in Caucasian patients with either ET or PMF. The large discrepancy may be attributed to the different frequencies of *JAK2*V617F, *MPL*, or *CALR* mutations, considering the unique genetic variation patterns of the three genes in different centers. Several of these differences may reflect the different sensitivities of the molecular approaches adopted, but the genetic and geographical differences by which ET and PMF develop may play more important roles. Genetic and geographical differences may imply that one or more undiscovered mutations are responsible for ET or PMF in a considerable proportion of patients with Chinese descent. Moreover, in recent years, a number of novel mutations including *TET2*, *IDH1/2*, *Ezh2*, *DNMT3A* and *Asxl1* have been described in *BCR-ABL1*-negative MPNs. None of these mutations were MPN-specific. They are more frequent in post-PV/ET MF, PMF and blast-phase MPN, and coexist with *JAK2*, *MPL* and *CALR* mutations, indicating that these abnormalities could involved in disease clonal evolution or blastic transformation of MPNs [18–20]. It is not clear as to whether these mutations contribute to molecular pathogenesis of triple-negative Chinese patients with MPN.

Currently, more than 50 different *CALR* indels have been described. In this study, we also reported 10 types of novel *CALR* indel mutations. All reported *CALR* mutations, including the 10 types of novel mutations, shifted the reading frame by one base pair and consequently produced distinct C-terminal amino acid sequences. Therefore, further studies are necessary to explain the generation of the unique mutation patterns of the *CALR* gene and to define whether these diverse *CALR* mutations can lead to different potential biological and clinical effects in MPN. Furthermore, in a study conducted in the Shanghai region of China [21], investigators found a number of scattered point mutations of the *CALR* gene in patients with and without the exon 9 indels. However, these somatic point mutations were not found in our center. Thus, we inferred that production of these point mutations may be closely associated with the environment in the Shanghai region.

Among all reported *CALR* indels, a 52 bp deletion (type 1 mutation) and a 5 bp insertion (type 2 mutation) were found in more than 80% of all *CALR*-mutant patients. Interestingly, the frequency of type 1 mutation was significantly higher in PMF than that in ET, suggesting a specific role of this mutation in myelofibrotic transformation [15,22]. This phenomenon is highly similar to that of the *JAK2*V617F mutation. Previous studies showed that *JAK2*V617F mutation is found in the majority of patients with PV and in many cases of ET or idiopathic myelofibrosis [1,2]. Several studies on biological function also confirmed that *JAK2*V617F mutation transfected into murine bone marrow cells produces erythrocytosis and subsequent myelofibrosis in recipient animals [1, 23,24]. This finding suggests that the *JAK2*V617F mutation prefers the expansion of the erythroid lineage. To further explore this issue, we compared our data with those of the other centers. As shown in Table 2, our study recorded a high frequency of type 1 *CALR* mutation and a low frequency of type 2 *CALR* mutation in PMF compared with ET. Data from two Western countries also showed the same results [7,8]. By contrast, studies conducted by Li *et al*. and Qiao *et al*. [11,13], reported that that the frequency of type 2 mutation is significantly higher than that of type 1 mutation in PMF. Moreover, Chen *et al*. [14] reported that the frequency of type 1 mutation is significantly higher in ET. These conflicting results may be attributed to insufficient case numbers or unknown molecular mechanisms. Evidence necessary to explain such discrepancies is still lacking. The current data do not completely support previous results. Biological effects of type 1 and 2 mutations should be investigated by building murine models.

We compared the demographic and hematological characteristics of Chinese patients with ET or PMF according to their *JAK2*V617F and *CALR* genotypes (Table 3). ET patients carrying a *CALR* indel displayed unique hematological phenotypes that differed from those with *JAK2*V617F mutations, in particular, the high platelet counts, and low leukocyte counts and hemoglobin levels. These findings are almost identical to those of many other studies [7,8, 11–14,16,17] and indicate that *CALR*-mutated ET appears to be substantially different from *JAK2*-mutated ET in terms of biological and hematological features. In contrast to the other studies [7,8, 11–14,16,17], no predilection toward the male sex or a younger age in *CALR*-mutated ET patients was observed. This difference in demographic parameters is probably associated with the different case numbers. For PMF patients, significant differences were not found between Chinese patients with PMF with mutant *CALR* and those with *JAK2*V617F in terms of gender, age, leukocyte counts, platelet counts, or hemoglobin levels. By contrast, Rumi *et al*. [15] showed a younger age predilection, lower leukocyte counts, and higher platelet counts in a large number of *CALR*-mutated PMF patients (n = 140). The large discrepancy probably results from the small cohort sizes and the different ethnical backgrounds.

In addition, demographic and hematological characteristics of ET patients with different *CALR* type 1 and *CALR* type 2 mutations were analyzed (Table 4). Some differences were mainly observed in our data. Patients with *CALR* type 1 mutation or *CALR* type 2 mutation respectively share a characterization with lower leukocyte counts than those with *JAK2*V617F mutation. Moreover, female patients with *CALR* type 1 mutation and female patients with *CALR* type 2 mutation also both presented lower hemoglobin levels, compared with female patients with *JAK2*V617F mutation. These observations indicate that *CALR* mutation and *JAK2*V617F mutation exert different effect on leukocyte and erythroid cell. Patients with *CALR* type 2 mutation displayed higher platelet counts than those with *JAK2*V617F mutation. However, patients with *CALR* type 1 and *CALR* type 2 mutations displayed the similar leukocyte counts, hemoglobin levels, and platelet counts. Similar results were presented by Qiao *et al*. [13] but not evaluated by others. These findings indicate that patients with *CALR* type 2 mutation may present a phenotype associated with the preferential expansion of the megakaryocytic lineage. Overall, these results suggest that the disease biology varies considerably according to different genetic lesions.

LAP scoring is a useful tool for evaluating granulocyte activation. LAP, which was classically named as neutrophil alkaline phosphatase, is stored in secretory vesicles of circulating neutrophils. Acquisition of LAP enzymatic activity is a late event during myeloid maturation and indicates that the enzyme is expressed in mature neutrophils. Hence, LAP is an indispensable marker of functionally mature neutrophils [25–27]. Passamonti *et al*. [28] found that neutrophils carrying the *JAK2*V617F mutation expressed higher levels of LAP in all types of MPN than healthy controls, thus suggesting that *JAK*2V617F may constitutively activate granulocytes. In the present study, we compared LAP expression characteristics of the MPN patients who were categorized according to their *JAK*2V617F and *CALR* genotypes. Fig 1 shows that LAP scores were markedly elevated in all types of MPN patients with *JAK2*V617F mutation, but frequently decreased in MPN patients with *CALR* mutation. As mentioned earlier, the evident differences observed among the diverse genetic subtypes suggest that the granulocyte biology in MPN patients varies considerably according to different genetic lesions. Recent research [7,8] confirmed that JAK–STAT (Janus kinase–signal transducer and activator of transcription) signaling is involved in the cytokine-independent growth of mutant *CALR*-expressing Ba/F3 cells. Seido oku *et al*. [26] confirmed that *JAK2*V617F signaling stimulates LAP expression in neutrophils, specifically by activating STAT3-dependent signaling pathway in vitro. *JAK2*V617F signaling promotes cell proliferation through STAT5 activation [26]. However, findings of this study also indicate that mutant calreticulin likely results in unknown abnormalities in the metabolic pathways of neutrophils.

In *JAK2*V617F-positive MPN patients, we found that LAP expression was also distinct among disease categories. Highest LAP levels were found in patients with PV, followed by patients with PMF and then with ET. A similar observation was previously noted by Basquiera *et al*. [29]. Such finding is consistent with previous studies [26,28] wherein patients carrying homozygous mutations exhibited higher levels of neutrophil LAP than those carrying heterozygous mutations, suggesting that LAP levels in neutrophils depend on the levels of *JAK2*V617F expression. Homozygous mutation for *JAK2*V617F is more frequent in PV than in ET or PMF. Thus, the highest LAP score in patients with PV may be due to a greater prevalence of homozygosity for *JAK2*V617F mutation in PV. Overall, our results strengthen the findings previously reported by other groups.

In conclusion, we reported a relatively lower mutation rate of *CALR* gene and a higher triple-negative frequency in Chinese MPN patients than in Caucasian patients. These data imply that a genetic alteration may be involved in up to 48% of triple-negative Chinese patients with ET or PMF. Hence, investigation of mutant molecules in our mutation-negative patients should be further investigated using exomic and whole genome sequencing. We also revealed that Chinese ET patients with *CALR* indels exhibited unique phenotypes similar to Caucasian ET patients, including low Hb levels, low WBC counts, and high platelet counts. Our data showed that patients with *CALR* type 2 mutation may present a phenotype associated with the preferential expansion of the megakaryocytic lineage, which is in contrast to those with *CALR* type 1 mutation. This study is the first to report that patients carrying *CALR* indels have relatively low LAP expression, which is a unique phenotype that is discrepant from those with *JAK2*V617F.
