# Correction: Frequencies, Laboratory Features, and Granulocyte Activation in Chinese Patients with *CALR*-Mutated Myeloproliferative Neoplasms

**DOI:** 10.1371/journal.pone.0141173

**Published:** 2015-10-15

**Authors:** Haixiu Guo, Xiuhua Chen, Ruiyuan Tian, Jianmei Chang, Jianlan Li, Yanhong Tan, Zhifang Xu, Fanggang Ren, Junxia Zhao, Jie Pan, Na Zhang, Xiaojuan Wang, Jianxia He, Wanfang Yang, Hongwei Wang

There are errors in the Funding section. The correct funding information is as follows:

This work was supported by National Natural Science Fund (NO. 81241014), Shanxi Natural Science Fund (NO. 2011011038–2), The Science and Technology Innovation Fund of Shanxi Medical University (NO. 01201413), The Younger Fund of the Second Hospital of Shanxi Medical University (NO. 20120101), The Graduate excellent innovation Fund of Shanxi Province (NO. 20143101). The funders had no role in study design, data collection and analysis, decision to publish, or preparation of the manuscript.
